# Proline-Rich Region II (PRR2) Plays an Important Role in Tau–Glycan Interaction: An NMR Study

**DOI:** 10.3390/biom12111573

**Published:** 2022-10-27

**Authors:** Anqesha Murray, Lufeng Yan, James M. Gibson, Jian Liu, David Eliezer, Guy Lippens, Fuming Zhang, Robert J. Linhardt, Jing Zhao, Chunyu Wang

**Affiliations:** 1 Center for Biotechnology and Interdisciplinary Studies, Department of Chemistry and Chemical Biology, Departments of Biological Sciences, Rensselaer Polytechnic Institute, Troy, New York, NY 12180, USA; 2 Division of Chemical Biology and Medicinal Chemistry, Eshelman School of Pharmacy, University of North Carolina, Chapel Hill, NC 27514, USA; 3 Program in Structural Biology, Department of Biochemistry, Weill Cornell Medical College, New York, NY 10065, USA; 4 Toulouse Biotechnology Institute, CNRS, INRA, INSA, University of Toulouse, 31077 Toulouse, France; 5 College of Food Science and Nutritional Engineering, China Agricultural University, Beijing 100083, China

**Keywords:** Alzheimer’s disease, tau, heparin, proline-rich region

## Abstract

(1) Background: Prion-like transcellular spreading of tau pathology in Alzheimer’s disease (AD) is mediated by tau binding to the cell-surface glycan heparan sulfate (HS). However, the structural determinants for tau–HS interaction are not well understood. (2) Methods and Results: Binding-site mapping using NMR showed two major binding regions in full-length tau responsible for heparin interaction. Thus, two tau constructs, tau PRR2* and tau R2*, were designed to investigate the molecular details at the tau–heparin binding interface. The 2D ^1^H-^15^N HSQC of tau PRR2* and tau R2* lacked dispersion, which is characteristic for intrinsically disordered proteins. NMR titration of Arixtra into ^15^N-labeled tau R2* induced large chemical shift perturbations (CSPs) in ^275^VQIINK^280^ and downstream residues K281-D283, in which L282 and I278 displayed the largest shifts. NMR titration of Arixtra into ^15^N-labeled tau PRR2* induced the largest CSPs for residue R209 followed by residues S210 and R211. Residue-based CSP fitting showed that tau PRR2*–Arixtra interaction had a much stronger binding affinity (0.37–0.67 mM) than that of tau R2*–Arixtra (1.90–5.12 mM) interaction. (3) Conclusions: Our results suggested that PRR2 is a crucial domain for tau–heparin and tau–HS interaction.

## 1. Introduction

Alzheimer’s disease (AD) is characterized by amyloid plaque and neurofibrillary tangles (NFTs) in brain pathology. NFTs are composed of microtubule-associated protein tau (MAPT), the normal functions of which include bundling and stabilizing microtubules (MTs) in neurons. In AD, hyperphosphorylated tau dissociates from microtubule and aggregates to form NFTs. Recent evidence from cell cultures [1,2], animal models [3,4,5], and human pathology [6] showed that tau/NFTs spread through neural networks in an orderly and ‘prion-like’ manner mediated by transcellular movement of tau [7,8,9]. The transcellular movement of tau is facilitated by tau binding to cell-surface heparan sulfate proteoglycans (HSPGs) [10,11,12,13].

HSPGs are a diverse family of proteoglycans with heparan sulfate (HS) chains covalently linked to a protein core. HS is a linear, polyanionic glycosaminoglycan (GAG) composed of disaccharide repeats of uronic acid (glucuronic acid or iduronic) and glucosamine with sulfation substitution possible on the 3-OH, 6-OH, and -NH of the glucosamine residue and the 2-OH of the uronic acid residue. Prior studies have demonstrated that tau interaction with HSPGs depends on the GAG chain length and specific sulfation patterns [12,13,14,15]. Among the common sulfo groups, 6-O-sulfation is the most important for HS–tau interaction. Recently, the rare 3-O-sulfation was shown to play a crucial role in HS–tau interaction and cellular uptake of tau [16,17]. HS and heparin GAG chains have overlapping structures with heparin being more highly sulfated; thus, heparin is often used as a stand-in for HS in binding studies (Capila and Linhardt, 2002). 

**Figure 1:**
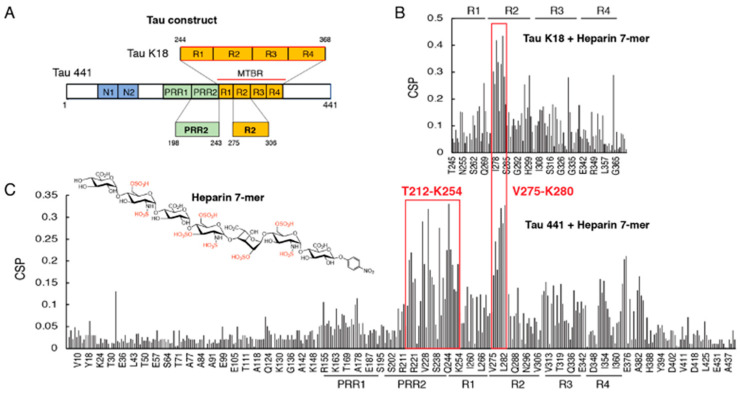
Chemical shift perturbations (CSPs) of tau K18 and tau 441 titrated by heparin 7-mer. (**A**) The constructs for tau 441 (full-length tau) and tau K18. Residue numbering is based on the numbering of tau 441. (**B**) Bar graph of ^1^H-^15^N residue chemical shift perturbations (CSPs) of tau K18 titrated with heparin 7-mer (1:1). (**C**) Bar graph of ^1^H-^15^N residue chemical shift perturbations (CSPs) of tau 441 titrated with heparin 7-mer (1:0.5). Subdomains PRR1, PRR2, R1, R2, R3, and R4 are indicated in the graph. The chemical structure of heparin 7-mer is shown in (**C**).

The primary sequence of the longest tau isoform htau40, which is composed of 441 a.a., features the N-terminal projection regions (N1 and N2), the proline-rich regions (PRR1 and PRR2), the microtubule-binding region (MTBR), and the C-terminal region (Figure 1A). The MTBR includes four internal repeat motifs (R1–R4), which mediate tau interactions with MTs [17,18] and other proteins [19], as well as tau aggregation [2]. The proline-rich region and MTBR have been established as major binding sites for heparin, a widely used HS analog [20,21]. Although tau–glycan interaction has been studied extensively, the details of the binding mechanism are still poorly understood. Detailed information at the tau–heparin interface is of fundamental importance in the understanding of the transcellular movement of tau in the pathogenesis of AD.

Here, by utilizing magnetic resonance spectroscopy (NMR), two major binding regions were determined in full-length tau as the major binding sites for heparin 7-mer. Thus, two new tau constructs were designed for these two binding regions, named PRR2* and R2* (* was used to distinguish the construct we designed from the PRR2 and R2 domains in full-length tau). NMR titrations were carried out on two new constructs individually, demonstrating the crucial role of PRR2 in tau–heparin interaction. Our work provides an important basis for further investigation to elucidate the molecular and structural mechanisms in tau–heparin interaction.

## 2. Materials and Methods

### 2.1. Materials

The overexpression and purification of the tau K18 and tau 441 proteins were performed as previously described [22,23]. Heparin with an average molecular mass of 15 kDa and polydispersity of 1.4 was purchased from Celsus Laboratories (Cincinnati, OH, USA), where it was extracted and purified from porcine intestine. Chemoenzymatic synthesis of heparin 7-mer was completed according to methods published previously [24,25]. Arixtra (fondaparinux sodium) was purchased from Sigma Aldrich.

### 2.2. NMR Titration of Tau K18 and Tau 441 with Heparin 7-Mer

The NMR spectrum of tau K18 and tau 441 were acquired at 10 °C on a Bruker 800 MHz NMR spectrometer equipped with a cryogenic probe. Aggregation did not occur at this low temperature. NMR data were processed and analyzed using Topspin 3.5pl7 (Bruker) and Sparky 3.115 (Goddard and Kneller, UCSF) [26]. ^15^N-labled tau K18 was dissolved in 100 mM NaCl, 10 mM Na_2_HPO_4_, and 4 mM DTT at pH 6.5 in 90/10% H_2_O/D_2_O. ^15^N-labled Tau 441 was dissolved in 25 mM NaH_2_PO_4_, 25 mM NaCl, 0.3 mM DTT, 2.5 mM EDTA, and 10% D_2_O at pH 6.5. ^1^H-^15^N HSQC spectra were recorded before and after addition of a 1:1 and 1:0.5 ratio of heparin 7-mer to tau K18 and tau 441, respectively. The normalized chemical shift perturbation (CSP) of tau for amide ^1^H and ^15^N chemical shifts upon heparin 7-mer addition were calculated using the equation $CSP=\sqrt{100\times \Delta H^{2}+\Delta N^{2}}$, where ΔH and ΔN are the differences between the chemical shifts of the free and bound forms of tau, respectively.

### 2.3. Reconstitution, Overexpression, and Purification of Tau PRR2* and Tau R2* Construct

Tau PRR2* (amino acid sequence _207_GSRSRTPSLPTPPTREPKKVAVVRTPPKSPSSAKSRLQTAPVPMPDLKNVK_257_) was inserted downstream of a small ubiquitin-like modifier (SUMO). Tau R2* (amino acid sequence _261_GSTENLKHQPGGGKVQIINKKLDLSNVQSKCGSKDNIKHVPGGGS_305_) were inserted downstream of the maltose binding protein (RBP) and fused with a sequence that harbored a thrombin cleavage site. Using the pETM41 vector for R2* and pET-21b(+) for PRR2*, the SUMO--PRR2*-His_6_ and His_6_-MBP-R2* fusion protein was successfully overexpressed in *E. coli* BL21(DE3) cells. Cells were lysed and the fusion protein was purified using a HisTrap™ Ni-NTA column (GE Healthcare). The tau R2* fusion protein was cleaved using thrombin followed by a second nickel column and then collection of the flow-through. The tau PRR2* fusion protein was cleaved with Ubl-specific protease 1 (ULP1) followed by applying the SUMO protease cleavage mixture to the nickel column and then collecting the elution using imidazole. ^15^N-labeled samples were obtained by growing cells in M9 minimal medium with 1 g/L of ^15^NH_4_Cl and 4 g/L of glucose. ^15^N and ^13^C doubly labeled tau PRR2* and R2* samples were obtained by growing cells in 1 g/L of ^15^NH_4_Cl and 4 g/L of ^13^C_6_-d-glucose (Cambridge Isotope Laboratories, MA, USA).

### 2.4. NMR Assignment of Tau PRR2* and Tau R2*

All NMR experiments were recorded at 10 °C for R2* and 4 °C for PRR2* on a Bruker 800 MHz spectrometer equipped with a cryogenic probe. All spectra were processed with topspin 3.5pl7 (Bruker) and NMRPipe/NMRDraw. The assignment of tau PRR2* and R2* was based on a series of 3D spectra using a uniformly ^15^N,^13^C-labeled sample including HNCACB, CBCA(CO)NH, HNCO, and HN(CA)CO. ^1^H-^15^N HSQC experiments were recorded with 8 scans, a recycle delay of 1.0 s, 3072 (t_2_) × 200 (t_1_) complex data points, and a spectral width of 16 ppm in the ^1^H dimension and 22 ppm in the ^15^N dimension. HNCACB and CBCACONH were recorded with 8 scans, 3072 (t_3_) × 75 (t_1_) × 256 (t_2_) data points, and a spectral width of 10 ppm in the ^1^H dimension, 22 ppm in the ^15^N dimension, and 60 ppm in the ^13^C dimension. HNCO and HN(CA)CO were recorded with 8 scans and 32 scans, respectively; 3072 (t_3_) × 74 (t_1_) × 128 (t_2_) data points; and a spectral width of 10 ppm in the ^1^H dimension, 22 ppm in the ^15^N dimension, and 13 ppm in the ^13^C. Linear prediction and zero-filing were used to obtain complex data matrixes before Fourier transformation to improve resolution.

### 2.5. NMR Titration of Tau PRR2* and Tau R2* with Arixtra and Heparin

The NMR spectra of tau PRR2* were acquired at 4 °C and tau R2* at 10 °C on a Bruker 800 MHz NMR spectrometer equipped with a cryogenic probe. Aggregation did not occur at this low temperature. NMR data were processed and analyzed using Topspin 4.2pl7 and Sparky. ^15^N-labled tau PRR2* and R2* were dissolved in 100 mM of NaCl, 10 mM of Na_2_HPO_4_, and 4 mM of DTT at pH 6.5 in 90/10% H_2_O/D_2_O. A series of separate ^1^H-^15^N HSQC spectroscopy experiments were performed on a 0.08 mM tau R2* or 0.120 mM PRR2* sample by adding increasing amounts of Arixtra. Normalized chemical shift perturbation (CSP) of tau for amide ^1^H and ^15^N chemical shifts upon Arixtra addition: $CSP=\sqrt{100\times \Delta H^{2}+\Delta N^{2}}$, where ∆*H* and ∆*N* are the differences between the chemical shifts of the free and bound forms of tau, respectively. Residue-based binding affinity was calculated using ∆*N* or ∆*H*, ∆_obs_ = ∆_max_ {([P]_t_ + [L]_t_ + K_D_ − ([P]_t_ + [L]_t_ + K_D_)^2^ − 4[P]_t_ [L]_t_ ^1/2^}/2[P]_t_, where ∆_max_ is the maximum shift change on saturation, [P]_t_ is the total protein concentration, [L]_t_ is the total Arixtra concentration, and K_D_ is binding affinity. HSQC titrations of PRR2* and R2* by heparin were also attempted; however, the NMR samples quickly aggregated upon addition of a small amount of heparin, precluding the acquisition of more quantitative data.

## 3. Results

### 3.1. Proline-Rich Region II (PRR2) Experiences Largest CSP in Full-Length Tau Binding to Heparin 7-Mer

Binding sites of heparin were mapped on the repeat domain of tau in the form of tau K18 (with all 4 repeats) and full-length tau (tau 441) using NMR. Here, a highly sulfated heparan sulfate (HS) heptasaccharide (7-mer) was synthesized and used to mimic the highly sulfated domain of HS [15]. For tau K18, the largest CSPs were found in the R2 domain, especially for the hexapeptide 275VQIINK280 and the downstream residues (K281, L282, and D283) (Figure 1B), which was consistent with our previous results when using heparin as a titrating ligand [14]. Similar perturbations around the R2 region were also observed in full-length tau (Figure 1C). Notably, residues within and near the PRR2 region from T212 to K254 exhibited comparable CSPs to R2. In contrast, PRR1 only showed minimal CSP. These results indicated that in addition to R2, PRR2 was also involved in the binding of tau to heparin. The R3 and R4 regions exhibited different CSPs in tau K18 compared with tau441, indicating that as a truncated construct, tau K18 could not fully recapitulate the binding of the full-length tau to heparin. Tau441 is overall dynamic and its interaction with ligands might be coordinated by different domains. 

### 3.2. Design of Tau Constructs R2* and PRR2* and Their NMR Assignments

**Figure 2:**
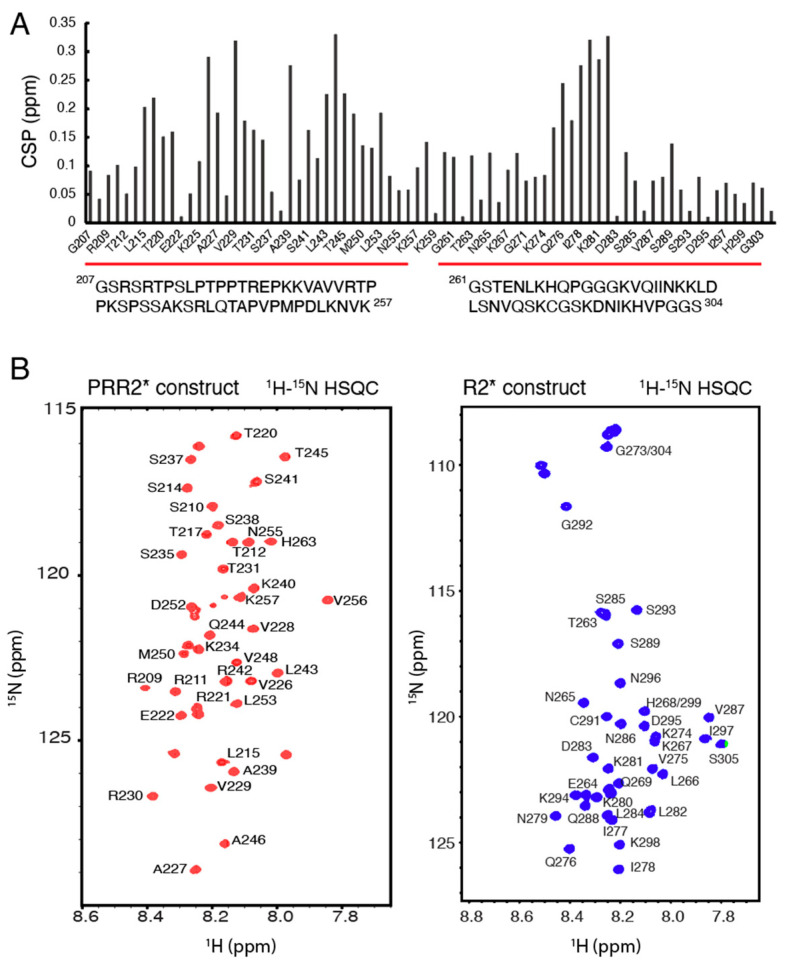
Tau PRR2* and R2* constructs. (**A**) Amino acid sequences and CSPs of tau PRR2* and R2*. (**B**) ^1^H-^15^N HSQC spectrum and chemical shift assignments of tau PRR2* and R2*.

Two new tau constructs were designed according to the CSPs (Figure 1) to further investigate the importance of PRR2 and R2 in recognizing heparin and HS. The construct containing residues from G207-K257 was named PRR2* and the construct containing residues from G261 to S304 was named R2* (Figure 2A). DNA plasmids for overexpressing PRR2* and R2* were designed and constructed as His-tagged SUMO fusion proteins or MBP fusion proteins with a thrombin cleavage site, respectively, using Genscript. These were then overexpressed in *E.coli* and purified with a nickel affinity column and thrombin digestion. ^1^H-^15^N HSQC spectra were recorded for isolated PRR2* and R2* (Figure 2B). NMR resonances were well resolved for both PRR2* and R2*; however, the narrow dispersion in the amide proton chemical shift was typical for an intrinsically disordered protein (IDP). For backbone NMR assignment of the two new constructs, a series of 3D NMR experiments were performed on ^13^C-^15^N doubly labeled samples, including HNCACB, CBCA(CO)NH, HNCO, and HN(CA)CO (data not shown). Amide assignments of PRR2* and R2* are shown in Figure 2B.

### 3.3. Characterization of R2*-Arixtra Interaction

**Figure 3:**
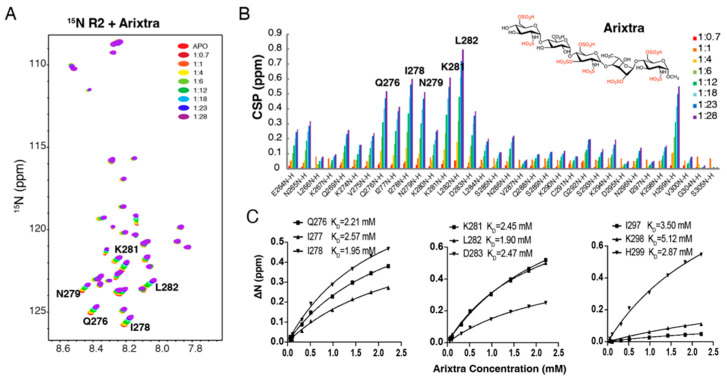
NMR titration of ^15^N labeled R2* by Arixtra. (**A**) Overlay of 1H-15N HSQC spectrum of 15N labeled tau R2* titrated by Arixtra at molar ratios from 1:0.7 to 1:28. (**B**) CSPs vs. residue number for tau R2* titrated by Arixtra at molar ratios from 1:0.7 to 1:28. (**C**) Residue-based affinities calculated from tau R2*–Arixtra titration. The chemical structure of Arixtra is shown in (**B**).

Next, we utilized the pentasaccharride Arixtra (fondaparinux sodium) to mimic the localized binding of heparin to tau. Arixtra is a synthetic ultralow-molecular-weight heparin widely used in the clinic for its anticoagulant activity. Because chain size is also an important determinant in tau–glycan binding [14], a much lower affinity was expected for tau–Arixtra interaction than for that of tau–heparin interaction. A series of 1H-^15^N HSQC spectra were recorded on ^15^N-labeled R2* by adding an increasing amount of Arixtra (from 1:0.4 to 1:25). As shown in Figure 3A, significant CSPs of R2* were observed with the addition of Arixtra. The largest CSPs were mostly localized to the hexapeptide 275VQIINK280 and downstream residues K281-D283. L282 and I278 displayed the largest shifts. The CSP pattern of R2* was consistent with that in tau K18 and full-length tau when titrated using heparin [14], suggesting that the isolated R2* construct mirrored the behavior of the R2* domain in the tau K18 and full-length tau protein. Residue-based affinities were obtained by CSPs fitted against the titration ratios (Figure 3C), which showed mM binding affinities that ranged from 1.90 mM to 5.12 mM.

### 3.4. Characterization of Tau PRR2*-Arixtra Interaction

**Figure 4:**
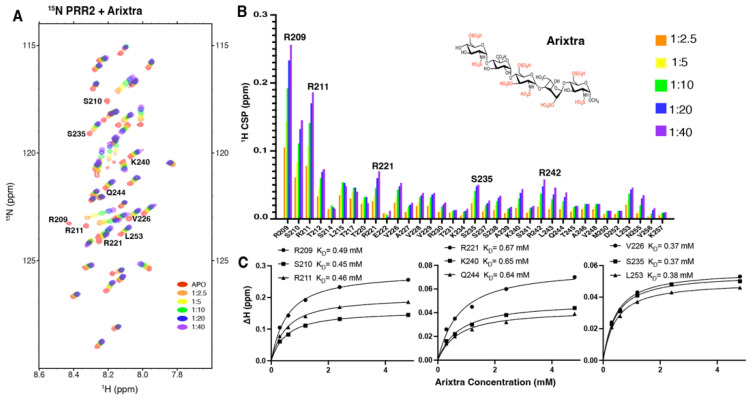
NMR titration of ^15^N tau PRR2* by Arixtra. (**A**) Overlay of ^1^H-^15^N HSQC spectrum of ^15^N labeled tau PRR2* titrated by Arixtra at molar ratios from 1:2.5 to 1:40. (**B**) CSPs vs. residue number for tau PRR2* titrated by Arixtra at molar ratios from 1:2.5 to 1:40. (**C**) Residue-based affinities calculated from tau PRR2*–Arixtra titration. The chemical structure of Arixtra is shown in (**B**).

For PRR2*, increasing amounts of Arixtra (from 1:0.5 to 1:8) were added into PRR2* and a series of ^1^H-^15^N HSQC spectra were recorded. As shown in Figure 4A, significant CSPs of PRR2* were observed with the addition of Arixtra. The largest CSPs were found for residue R209 followed by residues including S210, R211, R221, L215, T212, V226, K240, R242, and L243 (Figure 4B). Notably, N-terminal residues of PRR2*, R209, S210, and R211 dominated the CSPs caused by Arixtra, which was consistent with the electrostatic nature of tau–heparin binding as reported previously [14]. Residue-based affinities were obtained by CSPs fitted against the titration ratios (Figure 4C), which showed mM binding affinities that ranged from 0.37 mM to 0.67 mM. The binding affinities of PRR2*–Arixtra interaction were significantly higher than those of R2*–Arixtra interaction. R2 has been well established as an important binding site with dominating tau–heparin interaction [14,21]. Our results suggested that PRR2 is another crucial domain for tau–heparin interaction, if not more important than R2 for HS binding.

### 3.5. Characterization of PRR2*–Heparin and R2*–Heparin Interaction

**Figure 5:**
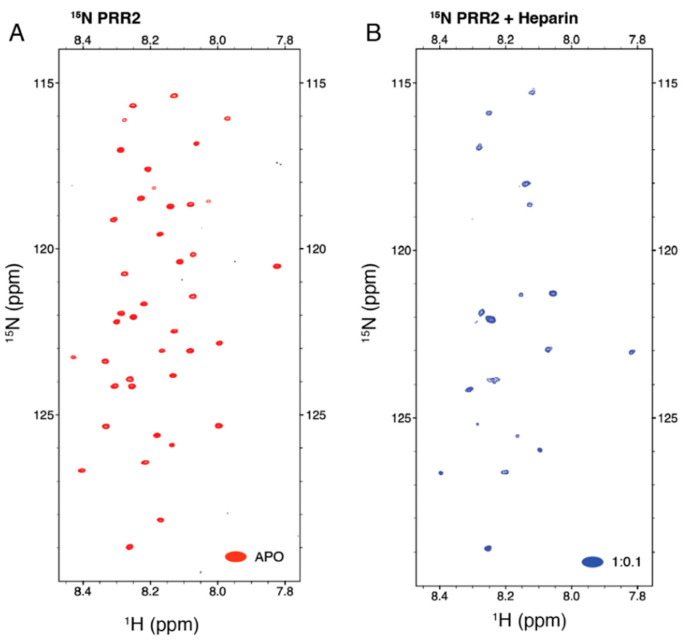
NMR titration of ^15^N-labeled tau PRR2* by heparin. (**A**) APO ^1^H-^15^N HSQC spectrum of ^15^N-labeled tau PRR2*. (**B**) ^1^H-^15^N HSQC spectrum of ^15^N-labeled tau PRR2* titrated by heparin at a molar ratio of 1:0.1 showing a prominent loss in peak intensity.

NMR titrations of ^15^N-labeled tau PRR2* by heparin were conducted to validate the role of the PRR2 domain in tau–heparin interactions. PRR2* titration by heparin at a molar ratio of 1:0.1 quickly led to sample precipitation and a decrease in the NMR signal of PRR2* (compare Figure 5A,B), precluding further and more quantitative analysis. A similar behavior was encountered in R2*–heparin titration. The large decrease in the peak intensity due to a small amount of heparin suggested that both R2* and PRR2* bound heparin with higher affinity than Arixtra.

## 4. Discussion

Although previous work on tau/HS interaction included PRR2 regions [20,21], quantitative binding studies of individual domains with glycan have not been carried out. Motivated by the large chemical shift changes observed in heparin and hepta-saccharide titration of tau441, we generated constructs for individual PRR2 and R2 domains (which were termed PRR2* and R2*). NMR titrations with a pentasaccharide Arixtra were then carried out that showed PRR2* bound Arixtra with a much higher affinity than R2*. In the residue-based affinity from fitting CSPs, the highest affinity for the PRR2* residue was 0.37 mM but was only 1.9 mM for R2*. Thus, PRR2 may have an even more important contribution to tau binding than R2, although R2 has been regarded as the prime site for glycan interaction, microtubule binding, and aggregation. When PRR* and R2* were titrated with heparin, both proteins quickly aggregated and precipitated, suggesting a much higher binding affinity to heparin than to the shorter Arixtra oligosaccharide. 

These data suggested that PRR2 plays a crucial role in HS binding. Full-length tau (htau441) interacted with heparin with a 10 nM K_D_ while K18 with all four repeat domains interacted with tau with μM affinity. The enhanced affinity in htau441 likely resulted from the additional interaction with PRR2. Because of the largely linear nature of both tau and HS, the binding affinity of K18 and PRR2 to HS is likely simply additive, giving rise to an increase in affinity of 2–3 orders of magnitude from additional glycan-binding sites in PRR2.

PRR2’s binding to glycan should be driven by electrostatic interactions. Out of 57 residues in PRR2*, there were 11 positively charged residues (Lys + Arg) and only 2 negatively charged residues (Glu + Asp). Thus, PRR2 was a highly positively charged patch in tau for the formation of favorable salt bridges with negatively charged sulfo groups in heparin and heparan sulfate.

As expected, there were a large number of proline residues in PRR2 (13) with multiple S/T-P motifs for tau phosphorylation. Proline isomerization has been reported to be important for controlling tau phosphorylation [27,28]. Because the transition from *cis* to *trans* will dramatically alter protein conformation, we speculated that proline isomerization may have an impact on tau–glycan interaction as well, which remains to be tested in our future work.

## 5. Conclusions

In summary, our results demonstrated the crucial role of the tau PRR2 domain in tau–heparin interaction, uncovering a structural requirement of tau recognition by heparin. This work increased our understanding of the mechanism of tau–heparin interaction and has important implications for PRR2 as a new target for interrupting the tau–heparin interface.

## Data Availability

Not applicable.
